# A mutation at IVS1 + 5 of the *von Hippel-Lindau *gene resulting in intron retention in transcripts is not pathogenic in a patient with a tongue cancer?: case report

**DOI:** 10.1186/1471-2350-13-23

**Published:** 2012-03-31

**Authors:** Takeshi Asakawa, Mariko Esumi, Sohei Endo, Akinori Kida, Minoru Ikeda

**Affiliations:** 1 Department of Otorhinolaryngology-Head and Neck Surgery, Nihon University School of Medicine, Tokyo173-8610, Japan; 2 Department of Pathology, Nihon University School of Medicine, 30-1, Ohyaguchikami-cho, Itabashi-ku, Tokyo 173-8610, Japan

## Abstract

**Background:**

Von Hippel-Lindau disease (VHL) is a dominantly inherited familial cancer syndrome predisposing the patient to a variety of malignant and benign neoplasms, most frequently hemangioblastoma, renal cell carcinoma, pheochromocytoma, and pancreatic tumors. VHL is caused by mutations of the *VHL *tumor suppressor gene on the short arm of chromosome 3, and clinical manifestations develop if both alleles are inactivated according to the two-hit hypothesis. *VHL *mutations are more frequent in the coding region and occur occasionally in the splicing region of the gene. Previously, we reported that the loss of heterozygosity (LOH) of the *VHL *gene is common in squamous cell carcinoma tissues of the tongue.

**Case Presentation:**

We describe a case of squamous cell carcinoma in the tongue caused by a point mutation in the splicing region of the *VHL *gene and discuss its association with VHL disease. Sequence analysis of DNA extracted from the tumor and peripheral blood of the patient with squamous cell carcinoma revealed a heterozygous germline mutation (c. 340 + 5 G > C) in the splice donor sequence in intron 1 of the *VHL *gene. RT-PCR analysis of the exon1/intron1 junction in RNA from tumor tissue detected an unspliced transcript. Analysis of LOH using a marker with a heterozygous mutation of nucleotides (G or C) revealed a deletion of the mutant C allele in the carcinoma tissues.

**Conclusions:**

The fifth nucleotide G of the splice donor site of the *VHL *gene is important for the efficiency of splicing at that site. The development of tongue cancer in this patient was not associated with VHL disease because the mutation occurred in only a single allele of the *VHL *gene and that allele was deleted in tumor cells.

## Background

Von Hippel-Lindau (VHL) disease has a prevalence of about 1 per 36,000 people with 20% of cases being sporadic and 80% familial [1,2]. The causal gene, *VHL*, is located on chromosome 3p26-25 and is known to act as a tumor-suppressor gene [3]. It has three exons that code for 213 amino acids and has functions to promote ubiquitination of hypoxia inducible factor 1- alpha [4]. There are characteristic abnormalities of the *VHL *gene in germline mutations from VHL disease and somatic mutations from sporadic cancers: more than a half of the former are missense mutations whereas 70% of the latter are frameshift mutations [5,6]. Thus, most pathogenic mutations are located in the coding region of the *VHL *gene. There are a few cases with splicing abnormality of the *VHL *gene and it is not yet clear whether an abnormal transcript of the *VHL *is pathogenic. In this report, we describe a splicing abnormality of the *VHL *gene in a patient with a squamous cell carcinoma (SCC) of the tongue.

## Case Presentation

The patient is a 55-year male with a tumor of the tongue, whose biopsy revealed a SCC. The cancerous tissue of the tongue along with peripheral blood were collected and stored at -80°C. Control material, both cancerous and non-cancerous, was obtained from a lingual cancer patient who did not have a *VHL *mutation. DNA was extracted from the tissue lysate using the phenol/chloroform method. DNA was extracted from whole blood as described previously [7]. Total RNA was prepared from cancerous tissue using Trizol (Invitrogen, Carlsbad, CA) according to the manufacturer's instructions. The study was approved by the Ethical Committee of Nihon University School of Medicine, conforming to the Helsinki Declaration. The patient gave his written informed consent to participate in this study and for publication of this case report.

**Figure 1:**
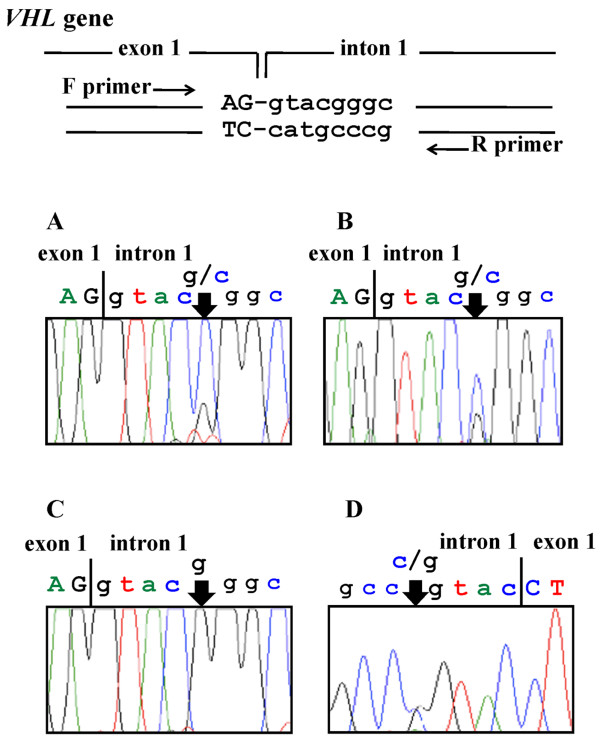
**Sequence profiles of exon 1 to intron 1 of the *VHL *gene**. The sequence of exon 1 to intron 1 of the *VHL *gene is shown in double- stranded form at the top. We compared DNA sequence from the cancerous tissue (A) and the peripheral blood (B) of the SCC patient to the non-cancerous tissue of a control patient (C). We observed a heterozygous c. 340 + 5 G > C point mutation of the *VHL *gene in the DNA from the cancerous tissue (A) and peripheral blood (B) of the patient with SCC of the tongue, but not in the control (C). The DNA from the peripheral blood of the patient was also sequenced in the reverse direction (D).

The three exons of the *VHL *gene, and their flanking regions, were amplified as described previously [6]. The polymerase chain reaction (PCR) product was purified with a Microcon 100 filter (Amicon, Inc., Beverly, Mass.) and sequenced using a BigDye Terminator Cycle Sequencing kit (Applied Biosystems, Foster City, CA). We determined nucleotide sequences using an ABI310 Genetic Analyzer. We observed a heterozygous c. 340 + 5 G > C point mutation of the *VHL *gene in the DNA from the cancerous tissue and peripheral blood of the patient with SCC of the tongue, but not in the control (Figure 1). The mutation is located at a splice donor site and could therefore lead to an unspliced transcription product.

To determine whether this had occurred, we amplified the transcription product using RT-PCR and performed real time PCR. Briefly, cDNA was synthesized from 2 μg of DNase I-treated RNA from tumor tissue using 50 pmol of random primers and 5 units of AMV reverse transcriptase version 2.2 (Life Sciences, St. Petersburg, Florida, USA). To identify the exon 1-intron 1 and exon 1-exon 2 regions, we performed SYBR Green PCR (Applied Biosystems) according to the manufacturer's instructions using intron primers E1/I1F (5'-CACAGCTACCGAGGTACCG-3') and I1R (5'-GAATGCTCTGACGCTTACGA-3'), and exon primers 1/2 F (5'-AGCTACCGAGGTCACCTTTG-3') and 2/3R (5'-CAGAGTATACACTGGCAGTG-3'), respectively. The amplifications were carried out by 40 cycles at 95°C for 15 sec and 60°C for 60 sec using an ABI7000 (Applied Biosystems). As a control, we used glyceraldehyde 3-phosphate dehydrogenase (*GAPDH*) gene primers (5'-GGTCGGAGTCAACGGATTTG-3' and 5'-GCATCTCGCTCCTGGAAGAT-3'). PCR products were run on 2% agarose gels to confirm fragment sizes.

**Figure 2:**
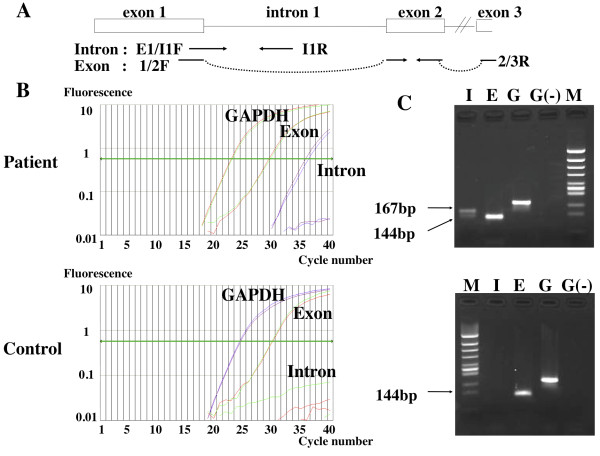
**RT-PCR of *VHL *transcripts. A**. Primer positions of intron and exon PCR. **B**. Amplification plots of real-time PCR. We performed real-time PCR for cDNA from the cancerous tongue tissue from the patient and cDNA from non-cancerous tissue of the control. *GAPDH *mRNA was quantified as an internal control. **C**. Agarose gel electrophoresis of real-time PCR products. The PCR product obtained from (B) was run on a 2% agarose gel for electrophoresis. Lane I, intron PCR (167 bp); lane E, exon PCR (144 bp); lane G, *GAPDH *PCR; lane G (-), *GAPDH *PCR using DNase-treated RNA without reverse transcription as a template; lane M, *Hin*cII digests of φx174 phage DNA.

As shown in Figure 2C, in our patient, a product of 167 bp was obtained by RT-PCR of tumor RNA with the exon 1/intron1 primer pair (E1/I1F and I1R), indicating that splicing of intron 1 had not occurred, at least in a portion of *VHL *transcripts. We confirmed that the transcript contained the intron sequence by direct sequencing (data not shown). Analysis of cDNA from the control patient did not produce a similar length transcription product. We ensured that the result from our intron RT-PCR was not a signal from contaminating DNA by treating the total RNA samples with DNase I prior to cDNA production (Figure 2). Semi-quantitative analysis suggested that the relative amount of transcripts in tumor tissue exhibiting abnormal splicing of intron 1 was only 1.5% of that exhibiting normal splicing of exon 2 and 3.

**Figure 3:**
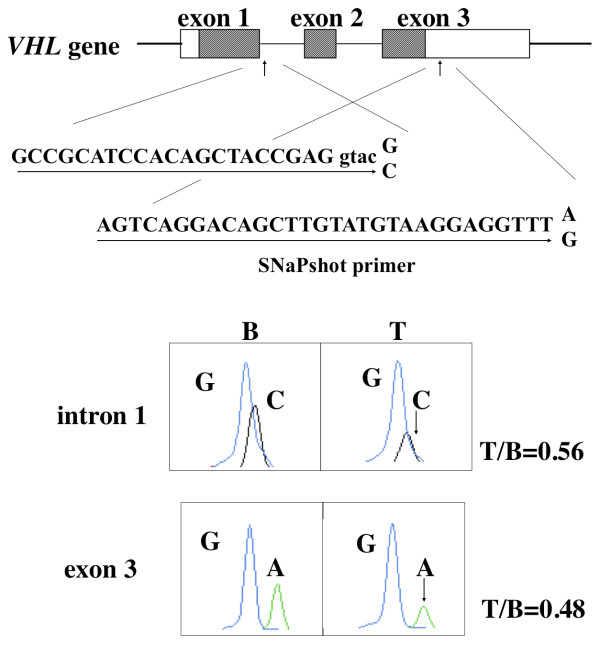
**LOH of the *VHL *gene**. LOH was analyzed at the heterozygous point mutation G/C of the intron 1 splice donor site and at the SNP A/G of the exon 3 of the *VHL *gene using a quantitative genotyping method. Comparison of cancerous tissue (T) and the peripheral blood (B) of the SCC patient indicated that the cancerous tissue had lost the mutant C allele (arrow) and the SNP A allele (arrow).

Then, loss of heterozygosity (LOH) of the *VHL *gene was determined by SNaPshot quantification as described previously [8]. Briefly, PCR was performed using two pairs of primers at the heterozygous point mutation G/C of the intron 1 splice donor site: E1/I1F (5'-CACAGCTACCGAGGTACCG-3') and I1R (5'-GAATGCTCTGACGCTTACGA-3') and at the SNP A/G of the exon 3: AGF (5'-CTGCCCATTAGAGAAGTATTT-3') and AGR (5'-AATTCCCACTGAATTACGTATA-3'). After purification using a Microcon 100 filter, the amplified product was subjected to a primer extension reaction using SNaPshot premix (Applied Biosystems) with the SNaPshot primers 5'-GCCGCATCCACAGCTACCGAGGTAC-3' and 5'-AGTCAGGACAGCTTGTATGTAAGGAGGTTT-3', respectively. The primer extension product was quantified using an ABI 310 genetic analyzer and GeneScan software (Applied Biosystems). The LOH analysis at the heterozygous point mutation (c.340 G > C) in intron 1 revealed that the tumor tissue had levels of the mutant allele that were about 50% lower than that observed in peripheral blood, suggesting loss of the mutant allele in tumor tissue (Figure 3). Thus, the reduction in intron transcription product was due to the loss of the mutant allele in the cancerous tissue.

As shown on Figure 1, we have found a germline point mutation, c.340 + 5 G > C, in the intron 1 splice donor site of the *VHL *gene of a patient with SCC of the tongue. We demonstrated that this base substitution was a mutation that produces a transcription product with abnormal splicing. This is the first report of abnormal splicing due to a mutation at the +5 position in intron 1 of the *VHL *gene. If the novel transcription product is translated without being spliced, then 35 amino acids and a stop codon would be read from intron 1. The C-terminally truncated polypeptide would compromise the elongin binding.

The consensus sequence of exon-intron junctions is 5'-A_64_G_73 _/G_100_T_100_A_62_A_68_G_84_T_63_-3' in which the subscripts indicate the percent occurrence of the specified base. U1 snRNA (3'-UCCAUUCAUApppGm-5') base pairs directly with the 5' splice site at the first stage of splicing. The extent of complementarity between U1 snRNA and the 5' splice site is 4-6 bp. The exon 1-intron 1 junction of the *VHL *gene is 5'-AG/GTACGG-3' in which the +4 and +6 positions of intron 1 are unpaired with U1 snRNA. Therefore, +5 position must be important to start splicing by base pairing with U1 snRNA.

A germline mutation at intron 1 splice site of the *VHL *gene has been found in numerous patients with VHL disease. Point mutations at IVS1 + 1 [9,10] or IVS1-1 [10], and an insertion or deletion at the IVS1 splice site [10,11] were found in several cases of VHL disease. The point mutation found here at IVS1 + 5 (G to C conversion) was present in cases of VHL disease type 1 [11-14]. Therefore, a point mutation at IVS1 + 5 can result in the abnormal splicing and are pathogenic. However, although there is abnormal splicing in the present case, the patient did not fulfill the current clinical diagnostic criteria of VHL disease [15] and had no family history of VHL disease. There are two possible interpretations for this apparent paradox. First, the patient has not yet developed VHL disease. Second, the mutation may not always be pathogenic and the patient will not develop VHL disease. Erlic *et al. *questioned the pathogenicity of the c.340 + 5 G > C mutation since it did not segregate with VHL in his family [16]. The question arises of whether the tongue cancer of the present patient is a VHL disease-associated cancer? The answer appears to be that it is not as there was no second hit from somatic cell mutation of the wild type *VHL *allele, although there was loss of the mutant allele in the tumor tissue. It is possible that the single hit allelic loss of the *VHL *gene increased the risk of cancer development. Recently, we observed frequent LOH of the *VHL *gene in sporadic tongue cancer but no somatic cell mutations such as missense, nonsense, and frameshift mutations [17]. LOH of several microsatellites on 3p was also detected in significant proportion of tongue cancer patients, and such deletions were often observed simultaneously across a large region of 3p within the same tumor sample [17]. The present patient showed LOH at all 5 informative microsatellites examined on 3p in addition to the *VHL *gene [17]. Thus, multiple tumor-suppressor genes on 3p may play a role in the development of tongue cancer in the present case.

## Conclusions

The fifth nucleotide G of the splice donor site of the *VHL *gene is important for the efficiency of splicing at that site. The development of tongue cancer in this patient was not associated with VHL disease because the mutation occurred in only a single allele of the *VHL *gene.

## Abbreviations

IVS: intervening sequence; VHL: *von Hippel-Lindau*; SCC: squamous cell carcinoma; PCR: polymerase chain reaction; GAPDH: glyceraldehyde 3-phosphate dehydrogenase; RT-PCR: reverse transcription-polymerase chain reaction; LOH: Loss of heterozygosity.

## Competing interests

The authors declare that they have no competing interests.

## Authors' contributions

SE and AK diagnosed the patient, collected the clinical data, and participated in the editing of the manuscript. MI participated in the editing of the manuscript. TA carried out the molecular genetic studies and drafted the manuscript. ME conceived of the study, participated in its design and helped to draft the manuscript. All authors have read and approved the final manuscript.

## Pre-publication history

The pre-publication history for this paper can be accessed here:

http://www.biomedcentral.com/1471-2350/13/23/prepub
